# Reduced Slow-Wave Rebound during Daytime Recovery Sleep in Middle-Aged Subjects

**DOI:** 10.1371/journal.pone.0043224

**Published:** 2012-08-13

**Authors:** Marjolaine Lafortune, Jean-François Gagnon, Véronique Latreille, Gilles Vandewalle, Nicolas Martin, Daniel Filipini, Julien Doyon, Julie Carrier

**Affiliations:** 1 Center for Advanced Research in Sleep Medicine, Hôpital du Sacré-Cœur de Montréal, Montréal, Québec, Canada; 2 Centre de recherche, Institut Universitaire de Gériatrie de Montréal, Montréal, Québec, Canada; 3 Département de psychologie, Université de Montréal, Montréal, Québec, Canada; 4 Département de psychologie, Université du Québec à Montréal, Montréal, Québec, Canada; Central Queensland University, Australia

## Abstract

Cortical synchronization during NREM sleep, characterized by electroencephalographic slow waves (SW <4Hz and >75 µV), is strongly related to the number of hours of wakefulness prior to sleep and to the quality of the waking experience. Whether a similar increase in wakefulness length leads to a comparable enhancement in NREM sleep cortical synchronization in young and older subjects is still a matter of debate in the literature. Here we evaluated the impact of 25-hours of wakefulness on SW during a daytime recovery sleep episode in 29 young (27y ±5), and 34 middle-aged (51y ±5) subjects. We also assessed whether age-related changes in NREM sleep cortical synchronization predicts the ability to maintain sleep during daytime recovery sleep. Compared to baseline sleep, sleep efficiency was lower during daytime recovery sleep in both age-groups but the effect was more prominent in the middle-aged than in the young subjects. In both age groups, SW density, amplitude, and slope increased whereas SW positive and negative phase duration decreased during daytime recovery sleep compared to baseline sleep, particularly in anterior brain areas. Importantly, compared to young subjects, middle-aged participants showed lower SW density rebound and SW positive phase duration enhancement after sleep deprivation during daytime recovery sleep. Furthermore, middle-aged subjects showed lower SW amplitude and slope enhancements after sleep deprivation than young subjects in frontal and prefrontal derivations only. None of the SW characteristics at baseline were associated with daytime recovery sleep efficiency. Our results support the notion that anterior brain areas elicit and may necessitate more intense recovery and that aging reduces enhancement of cortical synchronization after sleep loss, particularly in these areas. Age-related changes in the quality of wake experience may underlie age-related reduction in markers of cortical synchronization enhancement after sustained wakefulness.

## Introduction

Homeostatic modulation of sleep pressure has been studied in a variety of species, from fruit flies [1] to various mammals [2]–[4]. In mammals, more time awake produces higher cortical synchronisation during non-rapid-eye movement (NREM) sleep, whereas more time asleep is associated with lower synchronisation [5]. High levels of cortical synchronisation during NREM sleep is characterized by high-amplitude (>75 µV) electroencephalographic (EEG) slow waves (<4 Hz; SW). SW have two phases at the cellular level: a hyperpolarisation phase (surface EEG SW negative phase), during which cortical neurons are mostly silent, and a depolarization phase (surface EEG SW positive phase), during which most cortical neurons fire intensively [6], [7]. Animal studies have demonstrated that, under high homeostatic sleep pressure, NREM sleep is characterized by short periods of intense cortical neuronal firing (ON periods) which alternate frequently with periods of neuronal silence (OFF periods). Conversely, under low homeostatic sleep pressure, NREM sleep is characterized by longer ON periods interrupted by sporadic OFF periods. In addition, under higher homeostatic pressure, surface SW are associated with steeper slope and more synchronous recruitment of cortical neurons, i.e., less variable entry into the ON and OFF phases compared to lower homeostatic pressure [8]. In humans, higher homeostatic sleep pressure is associated with not only higher SW density and amplitude, but also shorter positive and negative phase durations, higher SW frequency, and steeper SW slope [9], [10]. More synchronous entry into the depolarization and hyperpolarisation phases at the cellular level under higher homeostatic pressure may underlie steeper SW slope and shorter surface EEG durations of SW negative and positive phases [11].

Increasing evidence suggests that cortical synchronization during NREM sleep depends not only on the number of hours of wakefulness preceding sleep but also on quality of prior waking activity [12]–[15]. For instance, daytime motor learning task and sensory stimulation increase slow-wave activity (SWA; spectral power between 0.5–4.5 Hz) during the subsequent sleep episode in brain areas involved in these tasks [14], [15]. According to the synaptic homeostasis hypothesis, cerebral plastic processes during wakefulness produce a net increase in synaptic strength in several brain circuits. NREM sleep oscillations, and SW in particular, downscale synaptic strength to a sustainable energy level, enabling efficient use of grey matter and new learning [16]. Thus, NREM sleep cortical synchronization appears to depend not only on wake/sleep duration [5], but also rely upon behavior and brain activation pattern during the day [12]–[15], [17].

**Table 1:** Polysomnographic variables for young and middle-age in both sleep condition.

	Young		Middle-aged		Age effect	Sleep condition effect	Age x sleep condition inter-action	
PSG variable	B	R	B	R	F (p)	F (p)	F (p)	Effect
Sleep latency	9.2 (5.8)	3.5 (3.6)	10.5 (6.6)	5.2 (5.2)	n.s.	F = 42.2 (p<0.00001)	n.s.	B > R
REM latency	78.4 (23.2)	66.9 (33.3)	77.3 (31.3)	60.2 (36.5)	n.s.	F = 7.0 (p<0.02)	n.s.	B > R
Sleep duration	437.3 (38.5)	377.9 (65.1)	425.9 (47.0)	332.0 (59.8)	F = 7.0 (p<0.02)	F = 88.8 (p<0.00001)	F = 4.5 (p<0.05)	B : Y = MA R : Y > MA
Sleep efficiency	91.1 (5.7)	79.8 (14.4)	87.6 (6.0)	69.3 (13.1)	F = 10.5 (p<0.01)	F = 90.5 (p<0.00001)	F = 5.1 (p<0.03)	Y > MA B > R
Stage 1 (%)	7.7 (3.7)	7.9 (5.4)	8.0 (3.4)	8.7 (3.7)	n.s.	n.s.	n.s.	-
Stage 2 (%)	59.8 (5.6)	56.5 (9.7)	66.2 (5.7)	65.3 (7.1)	F = 22.6 (p<.0001)	F = 6.1 (p<0.02)	n.s.	Y < MA B < R
SWS (%)	9.1 (6.4)	16.8 (9.8)	4.0 (4.0)	9.1 (7.3)	F = 15.1 (p<0.001)	F = 97.5 (p<0.00001)	F = 4.6 (p<0.04)	Y > MA B < R
Stage REM (%)	23.4 (4.9)	18.7 (6.7)	21.8 (4.3)	16.9 (6.3)	n.s.	F = 48.5 (p<0.00001)	n.s.	B > R
NREM (min)	301.9 (35.6)	276.5 (51.5)	295.1 (40.5)	250.0 (50.9)	n.s.	F = 30.1 (p<0.00001)	n.s.	B > R

Untransformed mean (standard deviation); Y: Young, MA: Middle-aged, B: Baseline nocturnal sleep, R: Daytime recovery sleep.

Considerable changes in cortical synchronization during NREM sleep occurs with aging, with a substantial reduction in SWS, an increase in lighter NREM sleep stages and significant decrease in SWA from age 20 to 60 years [18], [19]. Older subjects show not only lower SW amplitude but also lower SW density, especially in prefrontal/frontal brain areas [11], where SW originate more frequently [20]. In addition, older subjects demonstrate lower SW slope and longer SW positive and negative phases compared to young subjects, which may indicate that cortical neurons take more time to synchronously enter SW hyperpolarization and depolarization phases [11].

Whether sleep of older subjects is less sensitive to a modulation in the number of hours of wakefulness prior to sleep is still a matter of debate in the literature. A few studies showed lower rebound of SWA after sleep deprivation in older subjects than in young subjects, particularly in anterior brain areas [21]–[23]. In addition, amplitude of SWA dissipation across the night is reduced in older compared to younger participants [24]. However, other studies showed that a reduction in sleep pressure (i.e. after a nap or when sleep opportunities are enhanced) produces similar effects on NREM sleep synchronization in young and older subjects [25], [26].

Reduced cortical synchronization during NREM sleep may reflect age-related changes in the ability of the brain to adapt to new experiences (i.e. brain plasticity) and in the quality of waking experience (lower cognitive/sensory stimulation and/or physical exercise) [27]–[31]. Importantly, lower NREM brain synchronization may also lead to important functional consequences such as an enhanced vulnerability to external and internal disturbances. For instance, sleep in older subjects is particularly vulnerable to circadian phases of high wake propensity, which means that it is more difficult for older people to sleep at the ‘wrong’ circadian phase (e.g., in the daytime) [32], even after sleep deprivation [23]. The mechanisms underlying this age-related vulnerability remain unknown. We suggested that lower NREM sleep synchronisation in older subjects would not be able to “override” as efficiently the daytime circadian waking signal [23], [33].

The present study will determine whether similar accumulation of wakefulness duration leads to different increase in NREM cortical synchronization in young and middle-aged subjects. The topography of SW homeostatic modulation and SW age-related differences will also be assessed. We predict that middle-aged subjects will have lower rebound of NREM cortical synchronization (lower SW density, amplitude and slope), particularly in frontal areas, compared to young subjects. Finally, this study will evaluate whether age-related changes in NREM sleep cortical synchronization predicts the ability to maintain sleep during daytime recovery sleep.

## Results

### Sleep architecture

Compared to baseline sleep, sleep efficiency was lower during daytime recovery sleep in both age groups but this effect was more prominent in middle-aged subjects than in young subjects (significant interaction age group * sleep condition; see Table 1). On the other hand, SWS was higher during daytime recovery sleep compared to baseline sleep in both age groups, but this effect was weaker in middle-aged subjects than in young subjects (significant interaction age group * sleep condition). Compared to baseline sleep, daytime recovery sleep was associated with lower sleep latency, REM latency, sleep duration, stage 2 percent and REM percent (significant sleep condition effects). Finally, compared to the young participants, middle-aged subjects showed shorter sleep duration and higher stage 2 percent (significant age group effects).

### All-night SW variables

**Figure 1:**
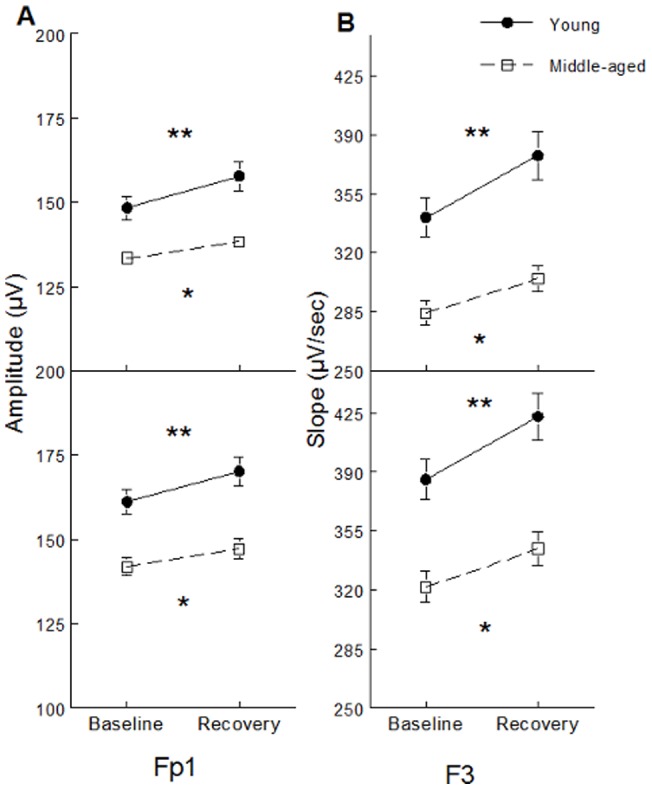
SW characteristics showing significant interactions between age group, sleep condition and derivations. SW amplitude (panel A) and SW slope (panel B) are shown for Fp1 (upper panel) and F3 derivations (lower panel) and for young subjects (black dots) and middle-aged subjects (open squares). Stars indicate differences between baseline sleep and daytime recovery sleep in young and middle-aged subjects (contrast analysis: *: p<0.0001; **: p<0.00001). A) Post-hoc analyses showed significant interactions between age group and sleep condition only in Fp1 (F(1,61) = 10.93, p<0.01) and F3 (F(1,61) = 7.11, p<0.01) derivations. B) Post-hoc analyses showed significant interactions between age group and sleep condition were found only on Fp1 (F(1,61) = 16.31, p<0.001) and F3 (F(1,61) = 8.19, p<0.01) derivations.

**Figure 2:**
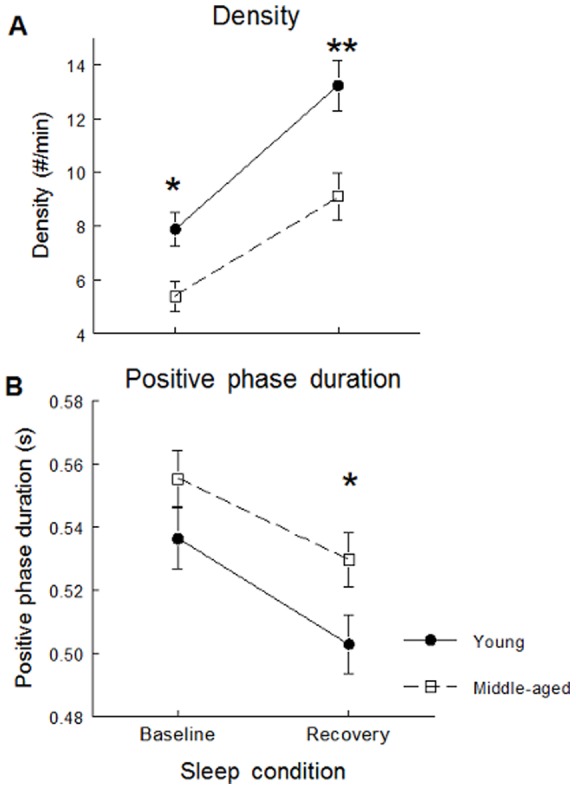
SW characteristics showing significant interactions between age group and sleep conditions. SW density (panel A) and SW positive phase duration (panel B) are shown for young subjects (black dots) and middle-aged subjects (open squares). Stars indicate differences between baseline sleep and daytime recovery sleep in young and middle-aged subjects (Contrast analysis: *: p<0.0001; **: p<0.00001).

**Figure 3:**
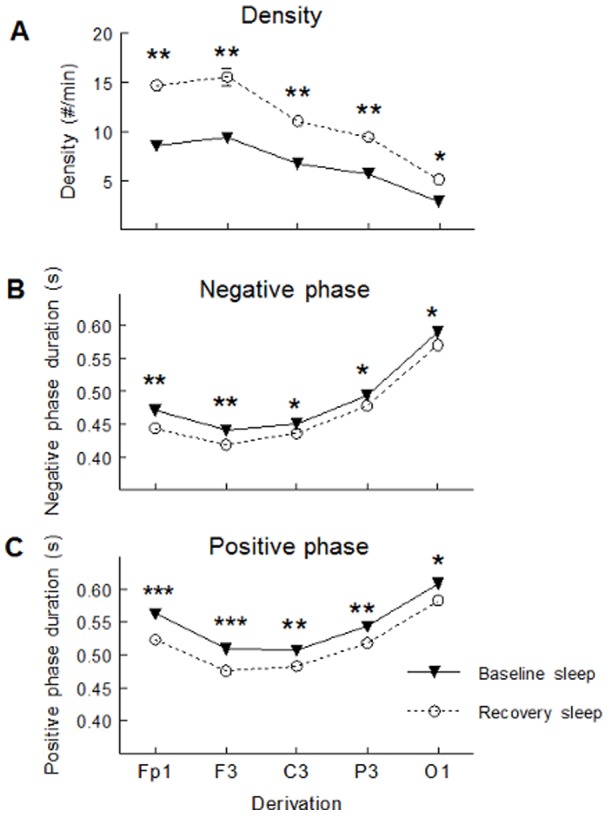
SW characteristics showing significant interactions between sleep condition and derivations. SW density (panel A), negative phase (panel B), and positive phase duration (panel C) are shown for baseline sleep (black triangles) and daytime recovery sleep (open circles). Stars indicate differences between baseline sleep and daytime recovery sleep for each derivation (Contrast analysis*: p<0.0001; **: p<0.00001; ***: p<0.000001).

SW slope was stronger and SW amplitude higher during daytime recovery sleep compared to baseline sleep but this effect was stronger in young subjects than in middle-aged participants in Fp1 and F3 derivations (significant interactions between age groups, sleep condition and derivation: F(4,244)>3.52; p<0.05, all cases; see Figure 1 for post-hoc and contrast analyses). Compared to baseline sleep, SW density increased and SW positive phase duration decreased during daytime recovery sleep in both age groups, but these effects were weaker in middle-aged than in young subjects (significant interactions between age group and sleep condition; F(1,61)>4.56, p<0.05; see Figure 2 for contrast analyses). Compared to baseline sleep, SW density was higher and negative and positive phase durations were shorter during daytime recovery sleep, and this effect was more prominent in anterior derivations compared to posterior derivations (significant interactions between sleep condition and derivations; F(4,244)>7.54, p<0.001; see Figure 3 for contrast analysis).

**Figure 4:**
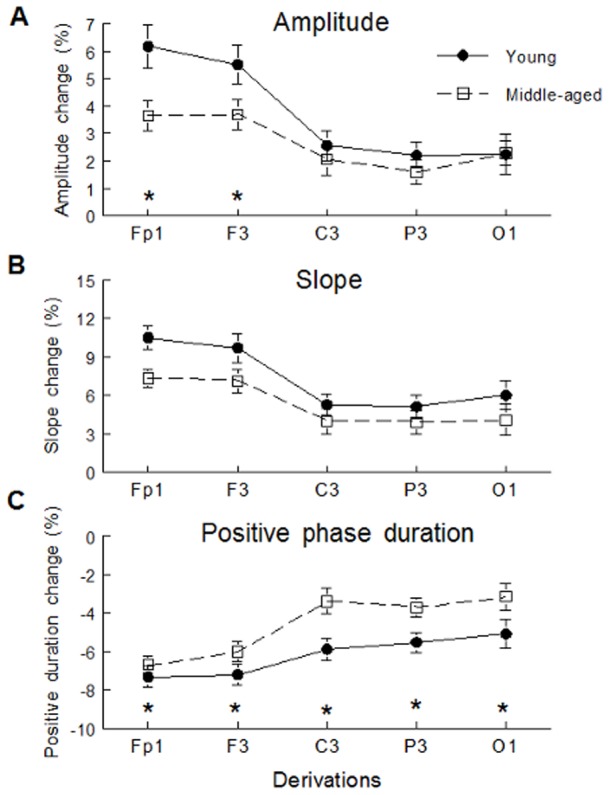
SW percent of change from baseline. SW amplitude (panel A), SW slope (panel B) and SW positive phase duration (panel C) are shown for young subjects (black dots) and middle-aged subjects (open squares). Stars indicate differences between young subjects and middle-aged subjects in each derivation (contrast analysis: *: p<0.05). A) Young subjects showed higher percent of increase in SW amplitude only in Fp1 (F(1,61) = 7.31, p<0.01) and F3 derivations (F(1,61) = 4.28, p<0.05). B) Effect of age group on percent of increase in SW slope tended to be significant. C) Percent of decrease in SW positive phase duration showed significant effect of age group.

Analyses on percent of change from baseline gave comparable results. Middle-aged subjects showed weaker percent enhancement in SW amplitude during daytime recovery sleep than young participants did but in Fp1 and F3 derivations only (interaction age group*derivation: F(4,244) = 3.21, p<0.05; see Figure 4A for contrast analysis). Percent of increase in SW slope during daytime recovery sleep tended to be stronger in young than in middle-aged subjects (age effect: p = 0.06; see Figure 4B). In addition, the diminution of positive SW phase duration during daytime recovery sleep was more pronounced in young than in middle-aged participants (age effect: F(1,61) = 6.22 p<0.05; see Figure 4C). On the other hand, percent of change in SW density from baseline to daytime recovery sleep did not show a significant age difference.

### SW variables for the first and the last NREMP

**Figure 5:**
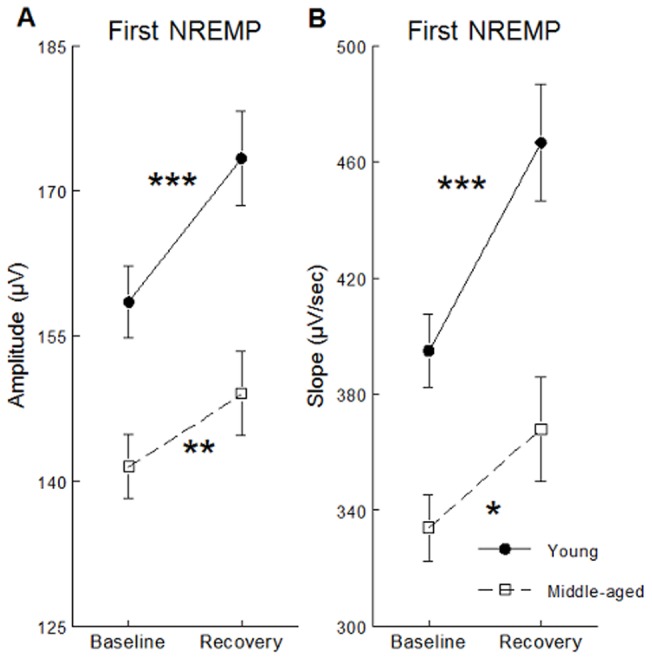
SW amplitude showing significant interaction between age group, sleep condition and NREMP. SW amplitude (panel A) and SW slope (panel B) are shown for young subjects (black dots) and middle-aged subjects (open squares), for baseline and recovery sleep during the first NREMP. Stars indicate differences between baseline and recovery sleep (Contrast analysis: *: p<0.05; **: p<0.001; ***: p<0.00001). A) After sleep deprivation, young subjects showed higher SW amplitude enhancement compared to middle-aged subjects during the first NREMP only (interaction age group * sleep condition: F(1,61) = 6.55; p = 0.05). B) After sleep deprivation, young subjects tended to show higher slope enhancement compared to middle-aged subjects during the first NREMP only (interaction age group * sleep condition: F(1,61) = 3,71; p = 0.06).

**Figure 6:**
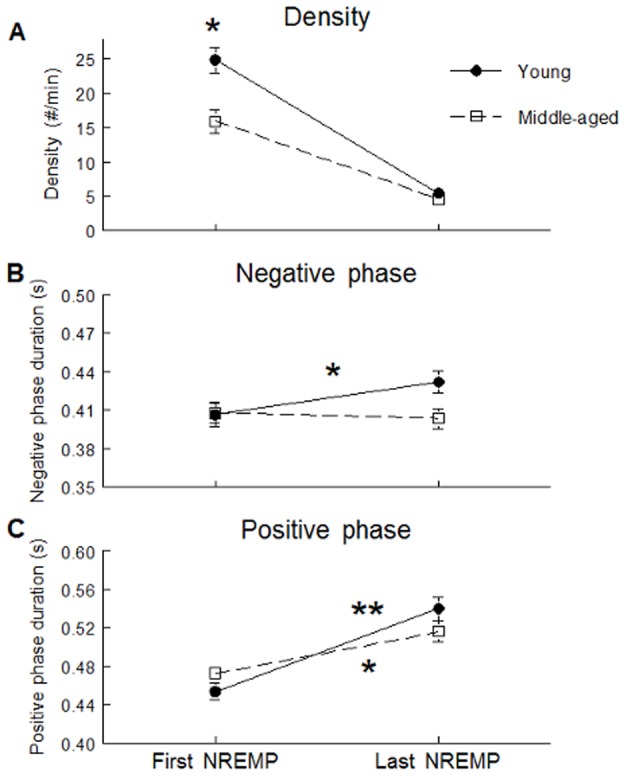
SW durations showing significant interactions between age group and sleep pressure. SW density (panel A), negative phase (panel B) and positive phase (panel C) on F3 derivation are shown for young subjects (black dots), middle-aged subjects (open squares) for the first and last NREMP averaged on the two sleep condition. Star indicates difference between young and middle-aged subjects for SW density and between the first and the last NREMP for SW durations (*: p<0.001; **: p<0.0001). A) Contrast analysis showed higher SW density in young than in middle-aged subjects during the first NREMP only (F(1,61) = 12.15; p<0.001). B) Contrast analysis showed significant SW negative phase duration increase between the first and last NREMP in young subjects (F(1,61) = 15.12; p<0.001) and no significant modification in middle-aged subjects. C) Contrast analysis showed higher SW positive phase duration increase between the first and last NREMP in young subjects (F(1,61) = 120.04; p<0.0001) compared to middle-aged subjects (F(1,61) = 37.48; p<0.001).

Analyses including the first and the last NREMP revealed that age differences in SW amplitude and SW slope enhancements after sleep deprivation were significant only during the first NREMP (interaction age group * sleep condition * NREMP: F(1,61)>4.09; p<0.05) see Figure 5A and Figure 5B for post-hoc and contrast analysis). Compared to the young, middle-aged participants showed lower SW density during both nights but this difference was significant only during the first NREMP (interaction age group * NREMP: F(1,61) = 12.54; p<0.001; see Figure 6A for contrast analysis). In addition, middle-aged subjects showed weaker SW negative and positive phase durations modulation between the first and the last NREMP, compared to the young (interaction age group * NREMP: F(1,61)>11.43; p<0.01; see Figure 6B and 6C for contrast analysis).

### Association between SW characteristics at baseline and change in sleep efficiency between baseline and daytime recovery sleep

Pearson correlations were performed between all-night SW variables during baseline sleep and change in sleep efficiency between baseline and daytime recovery sleep (absolute change and percent of change) in the young and the middle-aged groups separately and pooled together. No significant association was found (*R*<0.16; *p*>0.21).

## Discussion

The study aimed to compare the impact of 25-hours of wakefulness on NREM sleep cortical synchronization during a daytime recovery sleep episode in young and middle-aged subjects. The results support the notion that sleep deprivation elicits more neural cortical synchronization in anterior brain areas and that aging reduces enhancement of cortical synchronization after sleep deprivation particularly in these areas and in the first NREM period. However, NREM sleep neural synchronization did not predict the ability to maintain sleep during daytime recovery sleep.

As reported in previous studies in young subjects [9], [10], [34], compared to baseline sleep, SW slope was steeper, SW amplitude and density were higher and SW durations of positive and negative phases were shorter after sleep deprivation during recovery sleep in both age groups. These effects were more prominent in anterior derivations, supporting previous results using delta spectral power [21], [22], [35]. These results suggest that frontal brain areas elicit or may need enhanced cortical synchronization after sleep deprivation. Increasing evidence suggests that areas highly activated during wake generate more SWA than other brain areas [14], [15]. According to the synaptic homeostasis hypothesis, cerebral plastic processes during wakefulness produce a net increase in synaptic strength in several brain circuits. NREM sleep oscillations, and SW in particular, downscale synaptic strength to a sustainable energy level, enabling efficient use of grey matter and new learning [16]. The higher neural synchronization rebound after sleep deprivation in frontal areas may be explained by higher use and higher synaptic plasticity in those regions, which, in turn, will lead to higher increase in synaptic strength in frontal and prefrontal areas, compared to other brain region [14], [16], [36]. Interestingly, waking anterior brain areas activity also seems particularly sensitive to the effects of sleep loss. Hence, although brain glucose metabolism decreases globally during sustained wakefulness, this effect is more prominent in some cortical regions, like pre-frontal and frontal areas [37]–[39]. In addition, a number of cognitive functions associated with frontal lobes, such as executive functions, working memory, inhibition and mental flexibility are especially vulnerable to sleep deprivation [40]–[44].

**Figure 7:**
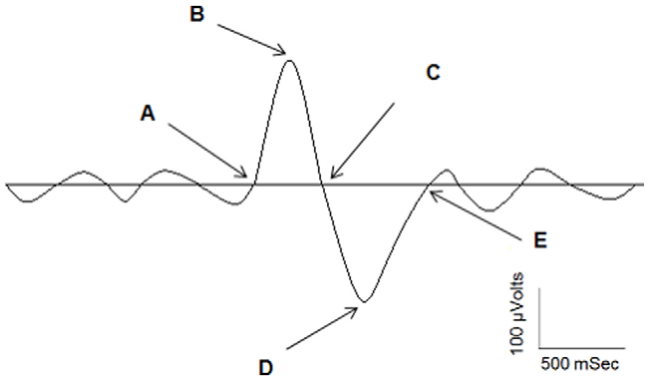
SW characteristics. SW frequency (number of cycles per sec), SW amplitude (difference in voltage between negative peak-B and positive peak-D of unfiltered signals expressed in µV), SW negative phase duration (number of sec between A and C), SW positive phase duration (number of sec between C and E), and SW slope between B and D expressed in µV/sec.

Middle-aged subjects showed lower cortical synchrony rebound after a 25-hours sleep deprivation compared to the young as indexed by SW density, SW positive phase duration, SW amplitude and SW slope. Furthermore, frontal dominance in SW amplitude and slope rebound was attenuated in older subjects compared to the young. This last results support a previous study showing lower delta spectral power enhancement in elderly subjects compared to young individuals after a 40-hour sleep deprivation [21], [22]. Whether a similar accumulation in the number of hours of wakefulness leads to a comparable increase in cortical synchronization in young and older subjects seems to depend on the amount of sleep pressure. Hence, studies showed lower delta spectral power rebound in older subjects after 25-hour and 40-hour sleep deprivation (high sleep pressure) [22], [23] whereas others depicted no age difference in SWA changes during low sleep pressure situations [25], [26]. In the present study, age differences in SW amplitude and slope enhancements after sleep deprivation were only significant at the beginning of the night, when homeostatic sleep pressure was at its highest.

Compared to young subjects, middle-aged subjects showed greater decrease in sleep efficiency after sleep deprivation during daytime recovery sleep, which replicate previous findings [23], [33]. Thus, older subjects have more difficulty in maintaining sleep during the high wake propensity circadian phase [32]. We proposed earlier that lower NREM sleep synchronization (lower SWS/SWA) associated with aging increases sleep disturbances caused by the daytime circadian waking signal [23]. The lower NREM sleep synchronization in middle-aged subjects would not be able to “override” the increasing circadian wake propensity as efficiently as in young subjects [23], [33], [45]. However, since none of the SW characteristics at baseline was associated with daytime recovery sleep efficiency in the present study, individual differences in NREM sleep synchronization does not predict the ability to maintain sleep during daytime.

Age-related changes in neural synchronization and in SW rebound may be associated with environmental and physiological differences in young and middle-aged subjects. Age-related decline in synaptic plasticity potential have been reported in animal [46], [47] and human studies [48]–[50]. Threshold for long term potentiation (LTP) in hippocampus increases, while it decreases for long term depression (LTD) in older rats compared to young rats. Moreover, LTP duration is shorter in older rats compared to young rats [46], [47]. In humans, different transcranial magnetic stimulation protocols that elicit LTP and LTD-like mechanisms in the motor cortex have been used in young and older adults [48]–[50]. Those studies showed an age-dependent reduction in motor cortex plasticity, measured by motor evoked potential. According to the synaptic homeostasis hypothesis, lower age-related synaptic plasticity would lead to lower increases in NREM neuronal synchrony after sleep deprivation via lower increases in synaptic strength during similar accumulation of wakefulness duration, particularly in frontal areas [36]. In addition to synaptic plasticity changes, age-related differences in the build-up of sleep pressure may also be associated with cognitive/sensory stimulation and physical exercise differences during the day [12]–[15], [17].

Whether vigilance in older subjects is less vulnerable to the effect of sleep deprivation remains a matter of debate. Whereas some studies have shown that reaction time in older subjects is less sensitive to the impact of sleep deprivation or the accumulation of wakefulness [51]–[56], other studies have not [57]–[59]. Lower reaction time decrement during sleep deprivation in older subjects may be related to a floor effect, because older adults show longer reaction time. In addition, some subjective variables (e.g., performance self-evaluation and sleepiness) appear to vary similarly in young and middle-aged subjects during sleep deprivation [54], [55], [59]. In a previous report (including 25 subjects of the present analyses), we found that middle-aged and young subjects showed similar time courses of subjective alertness and spectral power in theta/low alpha frequency bands during a 25-hour sleep deprivation [60]. We concluded that vigilance in middle-aged subjects is as sensitive to the accumulation of wakefulness impact of sleep deprivation as in younger subjects. Unfortunately, 24 of the 38 subjects added to the present report were not evaluated for baseline vigilance (e.g., they arrived at the laboratory only at the end of the afternoon for the sleep deprivation), limiting new analyses of vigilance. Nevertheless, combined with the present result, the results suggest dissociation in middle-aged subjects between sensitivity of both alertness and sleep to the number of hours of wakefulness.

In conclusion, our results have shown that the build-up of homeostatic sleep pressure is different in young and middle-aged subjects, particularly in anterior brain areas. Similar accumulations of wakefulness duration, in a context of high sleep pressure, lead to lower neural synchronization enhancements in middle-aged subjects compared to young subjects. Future studies should investigate age-related physiological and waking experience differences that may account for age-related differences in SW rebound after sleep deprivation.

## Materials and Methods

### Ethics Statement

All subjects signed an informed consent form and received monetary compensation. All research studies in which the subjects participated were approved by the ethical committee of the Hôpital du Sacré-Coeur de Montréal.

### Subjects

Sixty-three healthy participants were separated into two age groups: young (n = 29; 15 women and 14 men; 20 to 38 y.o., mean  = 27 years; SD  = 5) and middle-aged (n = 34; 20 women and 14 men; 40 to 60 y.o., mean  = 52 years, SD  = 5). A homemade questionnaire and a semi-structured interview were used to exclude potential subjects who smoked, used medication known to affect the sleep-wake cycle, complained about their sleep-wake cycle or reported habitual sleep duration of less than 7 hours or more than 9 hours. Potential subjects with a history of psychiatric or neurological illness and those who performed night work or transmeridian travel three months prior to the study were also excluded. Blood sample analysis (complete blood count, serum chemistry including hepatic and renal functions, prolactine level, testosterone level in men, and estrogen, FSH and LH levels in women) and urinalysis results were evaluated by a physician and were used to rule out significant medical conditions. Peri-menopausal women and women using hormonal contraceptives or receiving hormonal replacement therapy were also excluded. Pre-menopausal women reported regular menstrual cycles (25–32 days) in the year preceding the study, had no vasomotor complaints (i.e., hot flashes, night sweats) and showed low FSH levels (<20 iU/L). These women started the laboratory sessions in the follicular phase of their menstrual cycle. All postmenopausal women reported an absence of menses in the past year, and FSH levels were above 20 iU/L.

Prior to data acquisition, subjects underwent a polysomnographic (PSG) adaptation and screening night, including nasal/oral thermistor and electromyogram (EMG) leg electrode recordings to screen for poor sleep efficiency and sleep disorders. The presence of sleep disturbances such as sleep apneas and hypopnoeas (index per hour >10) and periodic leg movements (index per hour associated with micro arousal >10) resulted in the participant's exclusion.

### Procedures

#### Polysomnographic recording

PSG recordings were obtained from studies conducted in our laboratory between 1999 and 2006 [23], [33]. Subjects initially came to the laboratory for a baseline nocturnal sleep episode. The following night, subjects were sleep deprived. A morning recovery sleep episode was initiated one hour after their habitual wake time (following 25 hours of wakefulness). Thirty-nine subjects (17 young and 22 middle-aged) stayed in the laboratory for the entire 25 hours whereas twenty-four subjects (12 young and 12 middle-aged) left in the morning after baseline sleep and performed their regular activities until the end of afternoon, at which point they came back to the laboratory. During the sleep deprivation episode in the laboratory, all subjects remained awake in a semi-recumbent position in dim light (<15 lux) until the next morning. All sleep episodes were free of active pharmacological manipulation but 24 subjects (12 young and 12 middle-aged) received two placebo pills before daytime recovery PSG (placebo condition of a caffeine study) [33]. Bedtime and wake time in the laboratory were determined using averaged regular schedules obtained from sleep diary entries recorded seven days prior to the subject's PSG recording. EEG electrodes were placed according to the international 10–20 system using a referential montage with linked ears, chin EMG and left and right EOG. PSG was recorded using a Grass Model 15A54 amplifier system (gain 10,000; bandpass 0.3–100 HZ). Signals were digitalized at a sampling rate of 256 Hz using commercial software (Harmonie, Stellate System). Sleep stages were visually scored on C3 in 20-s epochs on a computer screen (LUNA, Stellate System) according to standard criteria at the moment of the studies [61]. EEG artifacts were detected automatically and rejected from analysis [62]. Further artifacts were also eliminated by visual inspection.

#### Automatic algorithm detection

SW were automatically detected on left parasagittal derivations: Fp1, F3, C3, P3, and O1. Data were initially bandpass filtered between 0.3 and 4.0 Hz using a linear phase Finite Impulse Response filter (–3 dB at 0.3 and 4.0 Hz) according to published criteria [11], [63]. SW were detected on artifact-free NREM sleep using the following criteria: 1) negative peak <–40 µV; 2) peak-to-peak amplitude >75 µV; 3) duration of negative deflection >125 ms and <1500 ms; and 4) duration of positive deflection <1000 ms.

For each SW, a number of characteristics were derived (Figure 7). SW amplitude (difference in voltage between negative and positive peak (B and D) of unfiltered signal expressed in µV), SW negative phase duration (number of sec between A and C), SW positive phase duration (number of sec between C and E) and SW slope between B and D expressed in µV/s. Characteristics of SW were averaged over all-night NREM sleep. Change in percent from baseline to recovery sleep for each SW variable was also calculated. Finally, SW characteristics on F3 derivation were averaged for four NREM periods (NREMP): each first and last sleep period for both sleep episodes. NREMP were determined according to published criteria and lasted at least 15 minutes and were followed by a REM period lasting at least 5 min [5].

### Statistical analyses

Initially, three-way ANOVAs with two independent factors (2 age groups: young and middle-aged; two sex groups) and one repeated measure (2 sleep conditions: baseline and recovery sleep) were performed to assess whether there were significant interactions between age group and sex on PSG variables and SW characteristics for each derivation. No significant interaction between age group and sex was found. Data from men and women were pooled together. To verify that the effects reported in the present study did not differ between the two protocols, we performed mixed ANOVAs with two independent factors (protocol and age group) and one repeated measure (sleep condition) for PSG sleep variables and SW characteristics for each derivation separately. No significant interaction between protocol, age group, and sleep condition was found, except for SW positive phase duration in P3 (interaction between protocol, age groups and sleep condition: F(1,59) = 6.28; p<0.05). Only young subjects showed a significant interaction between protocol and sleep condition (F(1,27) = 7.44; p<0.05). In young subjects, SW positive duration on P3 decreased between baseline sleep and recovery sleep in both protocols. However, this decrease was more significant in the protocol in which subjects stayed in the laboratory during the entire sleep deprivation period (F(1,27) = 110.95; p<.000001) compared to the other protocol (F(1,27) = 27.96; p<.0001). In middle-aged subjects, SW positive phase duration on P3 decreased between baseline sleep and recovery sleep (sleep condition effect: F(1,32) = 51.91; p<.000001), and there was no significant protocol effect. Because only SW positive phase duration on P3 showed significant interaction between age group, protocol, and sleep condition, and presented no significant differences between protocols in both sleep conditions, data from the two protocols were pooled.

To evaluate the impact of age and sleep deprivation on sleep architecture, mixed ANOVAs with one independent factor (2 age groups: young and middle-aged) and one repeated measure (2 sleep conditions: baseline and recovery sleep) were performed on PSG sleep variables. Mixed ANOVAs with one independent factor (2 age groups: young and middle-aged) and two repeated measures (2 sleep conditions: baseline and recovery sleep; 5 derivations: Fp1, F3, C3, P3 and O1) were performed on all-night SW characteristics. In addition, mixed ANOVAs with one independent factor (2 age groups: young and middle-aged) and one repeated measure (5 derivations: Fp1, F3, C3, P3, O1) were performed on all-night SW change in percent from baseline sleep to recovery sleep. To evaluate age-related differences in SW rebound across NREMP, mixed ANOVAs with one independent factor (2 age groups: young and middle-aged) and two repeated measures (2 sleep conditions: baseline and recovery; 2 NREMP: first and last) were performed on F3 derivation. P values for repeated measures with more than two levels were adjusted for sphericity with Huynh-Feldt corrections, but original degrees of freedom are reported. Differences in main effects were assessed with post hoc Tukey HSD tests. Contrast analyses were performed when significant interactions were found. Finally, Pearson correlations were performed between all-night SW variables during baseline sleep and change in sleep efficiency between baseline and daytime recovery sleep (absolute change and % of change) in the young and the middle-aged groups separately and pooled together. Results were considered significant when *p*≤0.05.
